# Minoxidil Overdose: A Case Report and Review of the Literature

**DOI:** 10.7759/cureus.86505

**Published:** 2025-06-21

**Authors:** Masakazu Kakurai, Yoshihiro Moriyama

**Affiliations:** 1 Dermatology, Tsuchiura Kyodo General Hospital, Tsuchiura, JPN

**Keywords:** alopecia, androgenetic alopecia, minoxidil, oral minoxidil, topical minoxidil

## Abstract

Minoxidil is a potent vasodilator originally used to treat severe refractory hypertension. Hypertrichosis is a common side effect of minoxidil, which led to the development and marketing of topical preparations for treating androgenetic alopecia (AGA). Minoxidil is primarily used to treat AGA; however, its inappropriate use can cause systemic side effects. Herein, we present the case of a 61-year-old Japanese man with an overdose of oral minoxidil that led to hypotension, breathlessness, edema, weight gain, and facial flushing. As the vital signs and results of laboratory and imaging tests were generally normal, no treatment was initiated. Two weeks after the initial visit, the patient’s body weight decreased by approximately 10 kg, and the edema disappeared. To date, 14 patients have been reported to have developed systemic side effects following an overdose of minoxidil-containing medications. Our literature review identified that systemic symptoms related to minoxidil overdose occurred in 10 of 14 patients after ingestion of a topical formulation, three after tablet ingestion, and one after application of a large amount of topical solution to the scalp. All 14 patients experienced hypotension and/or tachycardia. These symptoms occurred within six hours of the ingestion of 100-3000 mg minoxidil. One of 14 patients presented with a complication of myocardial infarction, and no patient died. Interestingly, fluid therapy was ineffective in the treatment of hypotension related to minoxidil overdoses. Since the primary pathophysiological effect of minoxidil is a decrease in systemic vascular resistance by arteriolar vasodilation, peripherally acting alpha-adrenergic agonists, such as phenylephrine, midodrine, and norepinephrine, may be safer and more effective than dopamine and epinephrine.

## Introduction

Minoxidil was first introduced in the 1970s as a treatment for severe refractory hypertension, owing to its potent vasodilatory properties [1,2]. Common side effects include tachycardia, edema, and weight gain [1,2]. Hypertrichosis is a common side effect that has led to the development and marketing of topical preparations for the treatment of androgenetic alopecia (AGA) [1,2]. Moreover, recent studies have demonstrated the safety and efficacy of low-dose oral minoxidil for AGA [1]. However, the inappropriate use of minoxidil-containing medications can lead to life-threatening systemic side effects that may require admission to an intensive care unit [3-15]. Because of its rarity, the clinical characteristics and potential treatment strategies for minoxidil overdose have not been comprehensively reviewed. To clarify the clinical features and suggest appropriate treatment strategies, we present a case of systemic symptoms following a minoxidil overdose and review 14 previously reported cases including ours.

## Case presentation

**Figure 1:**
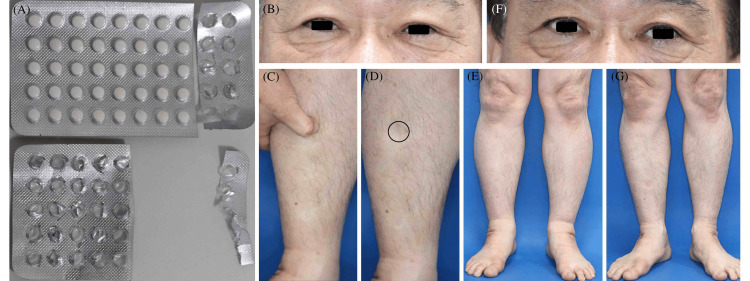
Clinical manifestations (A) He bit off a PTP of self-purchased minoxidil and ingested 35 tablets. (B-E) Facial edema (eyelid edema) and pitting edema of the lower legs (black circle) were observed. No significant edema was noted in the upper extremities or trunk. (F, G) Two weeks after the initial visit, neither facial nor lower leg edema was observed. PTP: press-through pack

A 61-year-old Japanese man was referred to our department for a suspected drug eruption. He had experienced facial flushing and edema for four days prior to presentation, followed by hypotension (96/58 mmHg) and breathlessness the day before presentation. His medical history included hypertension, hyperlipidemia, and AGA, and his medications included trichlormethiazide, amlodipine besylate, atorvastatin calcium hydrate, zolpidem tartrate, and minoxidil. The night before his symptoms began (five days before visiting our department), he was administered zolpidem tartrate. In a daze, he bit off a press-through pack (PTP) sheet of minoxidil that he had purchased and took 35 tablets (Figure 1A), corresponding to an ingested quantity of 350 mg of minoxidil.

On examination, he had an axillary body temperature of 36℃, a heart rate of 92 beats per minute, a blood pressure of 145/78 mmHg, and oxygen saturation of 95% on room air, without impaired consciousness. His body weight was 94.7 kg (weight measured within the last month was 83.0kg). Physical examination revealed facial edema without flushing and pitting edema of the lower legs (Figures 1B-1E). Edema was primarily noted in the eyelids and lower legs, but no significant edema was observed in the upper extremities or trunk. Blood tests showed elevated levels of C-reactive protein (0.79 mg/dL), creatine kinase (409 U/L), and N-terminal pro-brain natriuretic peptide (378 pg/mL). Other values, including white blood cell count, creatine kinase MB, and troponin T, were within normal limits (Table 1).

**Table 1:** Laboratory data of blood samples

Blood test	Reference value (male)	On arrival
White blood cell (/μL)	3300-8600	6950
Eosinophils (%)	0-7.0	1.9
Hemoglobin (g/dL)	13.5-17.0	14.0
Platelet (×10^4^/μL)	15.0-35.0	30.2
Total protein (g/dL)	6.6-8.1	6.4
Albumin (g/dL)	4.1-5.1	3.9
Aspartate aminotransferase (U/L)	13-30	25
Alanine aminotransferase (U/L)	10-42	46
Lactate dehydrogenase (U/L)	124-222	241
Sodium (mEq/L)	138-145	139
Potassium (mEq/L)	3.6-4.8	3.3
Urea nitrogen (mg/dL)	8-20	18
Creatinine (mg/dL)	0.65-1.07	1.18
C-reactive protein (mg/dL)	0-0.14	0.79
Creatine kinase (U/L)	59-248	409
Creatine kinase MB (U/L)	0-12	4
Troponin T (ng/L)	0-70	30
N-terminal pro-brain natriuretic peptide (pg/mL)	0-125	378
Free T3 (pg/mL)	2.30-4.00	3.14
Free T4 (ng/dL)	0.930-1.700	1.330
Thyroid-stimulating hormone (mIU/L)	0.610-4.230	1.670

No abnormal findings were noted on urine tests, electrocardiography (ECG), or chest radiography. Abdominal radiography revealed the absence of the PTP sheets. Cardiac ultrasonography revealed a left ventricular ejection fraction of 71%, grade I diastolic dysfunction in the left ventricle, and left atrial enlargement, but no evidence of right heart overload or pericardial effusion. Taken together, these findings were consistent with systemic effects of minoxidil overdose. Because vital signs and laboratory and imaging tests were generally normal, no pharmacotherapy was initiated. The patient was instructed to discontinue minoxidil and zolpidem tartrate. One week after the initial visit, the patient’s body weight decreased to 86.0 kg. Two weeks after the initial visit, it decreased to 85.1 kg, and the edema in the eyelids and lower legs disappeared (Figures 1F, 1G).

## Discussion

Minoxidil is a direct vasodilator introduced in the early 1970s to treat severe refractory hypertension [1,2]. The antihypertensive activity of minoxidil is due to its sulfate metabolite, minoxidil sulfate [2]. Minoxidil primarily acts by opening adenosine triphosphate-sensitive potassium channels in vascular smooth muscle cells, resulting in antihypertensive effects via vasodilation [2]. The main site of action of minoxidil is the artery. Arteriolar vasodilation caused by minoxidil stimulates the peripheral sympathetic nervous system via carotid and aortic baroreceptor reflexes, resulting in increased heart rate and cardiac output [2]. However, with long-term treatment, the heart rate and cardiac output tend to revert to pretreatment values [2]. 

The maintenance dose of minoxidil for the treatment of hypertension ranges from 10 mg to 40 mg daily, and side effects, including tachycardia, edema, and weight gain, occur in a dose-dependent manner [2]. Because hypertrichosis is also a common side effect among users, topical preparations were first marketed in the 1980s [1,2]. Minoxidil is used as a 2% and 5% topical solution to treat various alopecia conditions in males and females, although it is only approved for AGA [1]. Oral minoxidil is not recommended for the treatment of AGA in Japan. However, a recent study demonstrated that low doses of 2.5 mg or 5 mg daily are effective in treating AGA in males with minimal side effects [1]. Moreover, low doses between 0.25 and 1.25 mg daily have been shown to be safe and effective for treating AGA in females [1].

Although the safety and proper dosage of minoxidil-containing medications have been established, these medications are sometimes used inappropriately and can cause toxicity. To date, 14 patients, including the present patient, have been reported to develop systemic side effects due to an overdose of minoxidil-containing medications (Table *2*) [3-15].

**Table 2:** Reported cases of minoxidil overdose ECG: electrocardiography; M: male; F: female; NA: not available

Author/year	Age/sex	Formulation	Total dosage	Symptoms onset	Symptoms	ECG changes	Treatment	Course/complication
Isles et al. (1981) [3]	2/M	Tablets, 5 mg	100 mg	<1 hour	Tachycardia	None	None	Improved/none
Poff et al. (1992) [4]	20/F	Tablets, 10 mg	<650 mg	<90 minutes	Hypotension, tachycardia, nausea, and headache	ST depression and T-wave inversion	Fluid therapy and activated charcoal with sorbitol	Improved/none
MacMillan et al. (1993) [5]	52/M	Topical solution, 2%	1200 mg (17.6 mg/kg)	<2 hours	Hypotension, tachycardia, and disturbance of consciousness	ST depression	Fluid therapy, dopamine, phenylephrine, furosemide, and albumin	Improved/myocardial infarction
Farrell et al. (1999) [6]	26/F	Topical solution, 5%	3000 mg	<1 hour	Hypotension and tachycardia	ST depression and T-wave inversion	Fluid therapy, dopamine, phenylephrine, furosemide, activated charcoal with sorbitol	Improved/none
Garrard et al. (2011) [7]	48/M	Topical solution	NA	<80 minutes	Hypotension, dizziness, and syncope	NA	Fluid therapy, dopamine, phenylephrine, norepinephrine, and midodrine	Improved/none
Claudet et al. (2015) [8]	7/F	Topical solution, 5%	250 mg (11.4 mg/kg)	1 hour	Hypotension, tachycardia, and vomiting	T-wave flattening	Fluid therapy	Improved/none
Kikuchi et al. (2016) [9]	47/M	Topical solution	3000 mg	<4 hours	Hypotension, dizziness, and vomiting	None	Fluid therapy, dopamine, noradrenaline, and etilefrine	Improved/none
Gheshlaghiet al. (2018) [10]	61/NA	Topical solution, 5%	250 mg	<5 hours	Hypotension and chest pain	ST depression and elevation	Fluid therapy and activated charcoal with magnesium	Improved/none
Sinha et al. (2018) [11]	54/F	Topical solution	600 mg	NA	Hypotension, tachycardia, dizziness, nausea, headache, and chest pain	ST depression	Fluid therapy and phenylephrine	Improved/none
Dash et al. (2023) [12]	12/F	Topical solution, 5%	1000 mg	NA	Hypotension, tachycardia, giddiness, vomiting, and headache	ST depression and elevation and T-wave inversion	Fluid therapy, norepinephrine, and vasopressin	Improved/none
Chakar et al. (2023) [13]	36/M	Topical solution, 5%	500-1500 mg	6 hours	Hypotension, tachycardia, vomiting, abdominal and chest pain	ST depression and elevation and T-wave flattening	Fluid therapy, dopamine, and norepinephrine	Improved/none
Tripathee et al. (2024) [14]	17/F	Topical solution, 5%	3000 mg	NA	Hypotension, tachycardia, and chest pain	ST depression and T-wave inversion	Fluid therapy, phenylephrine, norepinephrine, midodrine, and furosemide	Improved/none
Ponomareva et al. (2024) [15]	23/M	Topical solution, 2%	Excessive application to the scalp	NA	Hypotension, dizziness, vertigo, blurred vision, and fatigue	None	None	Improved/none
Present case	61/M	Tablets, 10 mg	350 mg (4.2 mg/kg)	<6 hours	Hypotension, breathlessness, edema, weight gain, and facial flushing	None	None	Improved/none

Our literature review found that systemic symptoms due to minoxidil overdose occurred in 10 of 14 patients after ingestion of a topical formulation, three after tablet ingestion, and one after application of a large amount of topical solution to the scalp [15]. Ten were adults and four were children. Eleven patients overdosed accidentally, and the remaining three overdosed by suicide [4,6,9]. Among the 14 patients, all patients experienced hypotension and/or tachycardia. Other symptoms included dizziness, syncope, headache, vomiting, chest pain, edema, and weight gain. Although the dosage of minoxidil at which systemic symptoms appear may vary depending on specific factors (e.g., body weight and liver function), such symptoms have been reported following the ingestion of 100-3000 mg of minoxidil in a single dose. The present patient ingested 350 mg of minoxidil, which is equivalent to 70-140 times the recommended oral dose for the treatment of AGA in males. The fact that all reported patients developed symptoms within six hours of ingestion may be attributed to the pharmacokinetics of minoxidil, as peak blood concentrations are reached one hour after oral administration, and the serum half-life is three to four hours [2,5]. However, the hypotensive effects of minoxidil can persist for up to 72 hours, even at therapeutic doses, owing to the delayed formation of its active metabolites in the liver [2,5]. Therefore, as observed in the present patient, symptoms may persist for several days after ingestion of high doses.

Nine of 13 patients who underwent ECG showed transient ST-segment and/or T-wave changes, one of whom was diagnosed with unstable angina [10]. Additionally, one patient experienced myocardial infarction despite having no prior history of cardiac disease [5]. Transient or irreversible myocardial injuries are thought to be caused by a combination of increased myocardial oxygen demand due to tachycardia and decreased coronary perfusion secondary to systemic hypotension [7]. 

In our literature review, we identified that only one patient presented with a complication of myocardial infarction, and no patient died [5]. Twelve of 14 patients were initially treated with fluid therapy; however, this did not significantly increase blood pressure. Consequently, subsequent treatment involved the administration of vasopressors, such as dopamine, phenylephrine, midodrine, and norepinephrine. Given that the primary pathophysiological effect of minoxidil is a decrease in systemic vascular resistance owing to arteriolar vasodilation, the most recommended vasopressor is an agent with strong alpha-1 activation, such as phenylephrine, midodrine, or norepinephrine [5,7,8,13]. In contrast, the use of positive chronotropic drugs such as dopamine and epinephrine is not actively recommended as a treatment because of the potential for worsening tachycardia and increasing myocardial oxygen demand, which could lead to myocardial ischemia [5,7,8,13]. In the future, it will be important to educate users on the safe use of minoxidil, to collect and analyze reported cases in order to enable prompt responses to systemic side effects, and to establish more effective treatment strategies.

## Conclusions

To date, 14 patients have been reported to develop systemic side effects owing to an overdose of minoxidil-containing medications. Our literature review identified that systemic symptoms occurred in 10 of 14 patients after ingestion of a topical formulation, three after tablet ingestion, and one after excessive application of a topical solution to the scalp. All patients experienced hypotension and/or tachycardia. These symptoms occurred within six hours of ingestion of 100-3000 mg minoxidil. One of the 14 patients presented with a complication of myocardial infarction, and no patient died.

Fluid therapy was ineffective in the treatment of hypotension related to minoxidil overdoses. Since its primary pathophysiological effect is a decrease in systemic vascular resistance, peripherally acting alpha-adrenergic agonists, such as phenylephrine, midodrine, and norepinephrine, may be safer and more effective than dopamine and epinephrine. It is important to educate users on the safe use of minoxidil. Further studies are needed to establish more effective treatment strategies.
